# From mouse embryo assay to bovine embryo assay: towards an ethical and scientifically superior quality control standard in assisted reproduction technologies

**DOI:** 10.1007/s10815-025-03792-z

**Published:** 2026-01-30

**Authors:** Pilar Coy

**Affiliations:** 1 Department of Physiology, University of Murcia, International Excellence Campus for Higher Education and Research (Campus Mare Nostrum), Murcia, Spain 30100; 2 Institute for Biomedical Research of Murcia, IMIB-Pascual Parrilla, 30120 Murcia, Spain; 3 EmbryoCloud SL, Centre for Multidisciplinar Research in Biosciences, Espinardo Campus, 30100 Murcia, Spain

**Keywords:** Bovine embryo assay, Mouse embryo assay, Embryotoxicity, Assisted reproduction, Animal welfare, Regulatory standards

## Abstract

The mouse embryo assay (MEA) is the standard test used in assisted reproduction to evaluate the toxicity and effectiveness of culture media and consumables. However, the assay has been criticised for its limited sensitivity, inconsistencies between laboratories, and ethical concerns. Despite the 3Rs principles, over 111 million mice and rats were used in the USA in 2017, with an unknown proportion of these being used in the MEA. While the FDA has provided MEA guidelines, its aim is to phase out animal toxicity testing within 3–5 years. This article explores the possibility of replacing the MEA with the bovine embryo assay (BEA), providing justifications based on ethics, science, practicality, and economics. Through a review of MEA applications, market data, regulatory frameworks and industry disclosures, the article estimates the current impact of the MEA. Incorporating the BEA into regulations could eliminate the need to breed mice for the MEA and greatly reduce the use of animals. Standardising and validating the BEA would provide a reliable and ethically preferable alternative that aligns with the growing demand from regulators and society for non-animal testing methods.

## Introduction

It has been estimated that 111.5 million rats and mice were used for various research and toxicity control purposes in the USA alone in 2017 [1], even assuming the fulfilment of the principles of the 3Rs (Replacement, Reduction, and Refinement), developed over 50 years ago [2]. These principles are embedded in national and international legislation and regulations on the use of animals in scientific or regulatory procedures, as well as in the policies of organisations that fund or conduct animal research. While recognising the necessity of animal use in various fields, nine countries have incorporated language safeguarding animals into their constitutions, and the majority of countries currently have fundamental legal prohibitions against animal cruelty. Only eleven countries worldwide lack legislation to protect animals [3].

In this context, the widespread use of the mouse embryo assay (MEA) in the assisted reproduction industry, which is projected to reach USD 45.06 billion by 2026, is paradoxical. The MEA involves the sacrifice of mice and is the accepted method for assessing the toxicity and functionality of media, laboratory equipment, consumables, and any device that may come into contact with gametes or embryos. While the Food and Drug Administration (FDA) of the USA has issued guidance outlining recommendations for conducting the MEA to support premarket submissions and the release of device lots [4], the agency has also recently proposed the goal of phasing out animal toxicity testing within 3 to 5 years [5].

Different experimental work shows that the information provided by the MEA is equivalent to, or worse than, that provided by similar tests using livestock gametes collected at slaughterhouses [6]. These animals are not killed specifically for the purpose of the test, but rather for food. Furthermore, the metabolic differences between mice and human embryos are far greater than those between cattle and humans. This makes cattle a more appropriate model for testing the embryotoxicity of medical devices [7, 8]. 

Assuming this premise is valid, it can be deduced that incorporating the MEA test into the medical device regulatory framework is inappropriate. A similar argument can be made regarding the necessity of mouse breeding facilities worldwide for this purpose, as these would consequently become superfluous, representing an economic advantage. Furthermore, the substantial amount of waste generated by these facilities could be eliminated. This article provides substantiation for this perspective.

## Methods

A comprehensive search of the PubMed database using the search term “mouse embryo assay” yielded 48 articles that focused primarily on this test, which were then selected and analysed. Information on the medical devices (preferably culture media) used in assisted reproduction that had been tested by the MEA was gathered from company websites, advertising materials, instructions for use and certificates of analysis. To investigate the current legislation regarding the use of the mouse embryo assay in assisted reproduction in the EU, the websites of the European Medicines Agency (EMA), the European Commission and the European Society of Human Reproduction and Embryology (ESHRE) were explored. Legislation in China (National Medical Products Administration, NMPA), the USA (FDA), and Australia (Therapeutic Goods Administration, TGA) was also investigated.

Realistic estimates of the number of animals slaughtered for MEA purposes, the number of animal facilities involved in rearing these animals, and the amount of waste and residues generated were obtained through thorough searches of PubMed and specific websites. The same approach was used to gather data on the historical development of social movements committed to pursuing animal rights, as well as the ethical principles that became universal under the 3Rs premise.

## The field: assisted reproduction in numbers

According to the World Health Organization (WHO), approximately one in six people of reproductive age worldwide (17.5% of the adult population) will experience infertility at some point in their lives [9]. This is particularly prevalent in the context of common factors such as delayed age at first childbearing, obesity, sexually transmitted infections, smoking, endometriosis, polycystic ovary syndrome, and primary ovarian insufficiency.

Assisted reproductive technologies (ART) were developed to address many of the causes of infertility and have experienced explosive growth since 1978, the year when Louise Brown, the first baby born through in vitro fertilisation (IVF), was born [10]. Today, ART are used to treat various medical and genetic conditions, as well as for fertility preservation. Examples of ART procedures include pre-implantation genetic testing, oocyte, sperm, embryo and gonad tissue cryopreservation, endometrial receptivity analysis, and time-lapse imaging of embryos [11].

A generic search for the market size of ART yields data from various consultancies that currently place it at between $21.32 billion and $34.7 billion, with projected growth to over $40 billion from 2026 onwards in all cases (see Table 1).

**Table 1:** Estimates from various consultancies on the overall size of the assisted reproduction market and growth forecasts for the next few years

Source	Global Market Size calculated (year)	Global Market Size predicted (year)	CAGR*
Fortune Business Insights^1^	USD 21.32 billions (2018)	USD 45.06 billions (2026)	9.8%
GMI (Global Market Insights) ^2^	USD 34.7 billions (2023)	USD 62.8 billions (2032)	6.9%
Grand View Research ^3^	USD 25.7 billions (2022)	USD 41.4 billions (2030)	5.97%
EMR (Expert Market Research) ^4^	USD 29.23 billions (2023)	USD 82.39 billions (2032)	7.24%
Growth Market Reports ^5^	USD 26 billions 2022)	USD 43.5 billions (2031)	5.91%
Technavio ^6^	USD 22.5 billions (2018)	USD 62.3 billions (2028)	4.36%

^*^Compound annual growth rate

^1^https://www.fortunebusinessinsights.com/industry-reports/assisted-reproductive-technology-art-market-101811

^2^https://www.gminsights.com/industry-analysis/assisted-reproductive-technology-market

^3^https://www.grandviewresearch.com/industry-analysis/assisted-reproductive-technology-market

^4^https://www.expertmarketresearch.com/reports/assisted-reproductive-technology-market

^5^https://growthmarketreports.com/report/assisted-reproductive-technology-market-global-industry-analysis

^6^https://www.technavio.com/report/assisted-reproductive-technology-market-industry-analysis

Beyond its economic impact, ART’s social relevance and importance to human well-being are beyond question, as evidenced by the increasing number of IVF cycles performed by countries and the growing number of women attending infertility clinics for other treatments. In 2019, the European IVF Monitoring Consortium (EIM) reported 1,077,813 treatment cycles, representing a 7.0% (70,215 cycles) increase on the previous year. Since 1997, the growing number of clinics reporting to the EIM has led to a cumulative total of 12,804,411 treatment cycles and over 2,479,254 births [12]. In the USA, data from 2021 indicate that 86,146 infants (2.3% of all infants born in the USA that year) were conceived using ART [13]. In view of the extensive scope of the IVF process, it became apparent several years ago that a comprehensive and systematic approach was essential to assess the quality of each stage within the laboratory, and a whole world of regulations and recommendations began to develop.

## The past: quality management systems and regulatory issues in AR laboratories

“The implementation of suitable quality management systems (QMS) is not only a prerequisite for the accreditation of facilities but is also a fundamental aspect of achieving the desired outcomes” [14], whether this involves the birth of a healthy child, fertility preservation in oncology patients, or the treatment of reproductive pathologies. As Olofsson et al. [15] noted, a total quality management approach requires mapping all processes, fully describing the procedures involved, and defining performance targets for each procedure. Accordingly, multiple performance indicators for ART laboratories have been defined and are continually reviewed by international expert groups. Key outputs include the Vienna Consensus [16], which agreed on the definition, competence level, and benchmark values for 19 indicators, and the Cairo Consensus [17], which produced more than 50 guideline points covering embryo culture fundamentals, operational protocols, culture media, and laboratory equipment.

In parallel, the European Society of Human Reproduction and Embryology (ESHRE) launched initiatives aimed “at guaranteeing the implementation of good laboratory practice and defining the concept of qualified embryologists” [18]. A central effort was the publication of good practice guidelines for IVF laboratories. The 2015 edition [19] updated the 2008 version [18]. In Sect. 5.1, the following was established:
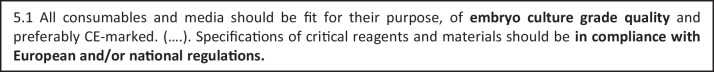


This statement has important regulatory implications for consumables and media in the EU market, yet the term “embryo culture grade” remains open to interpretation, potentially leading to variable regulatory outcomes.

Chronopoulou and Harper [20] highlighted regional differences in the regulation of IVF culture media across the EU, USA, Australia, and China. Since then, requirements have become more aligned, with a notable exception related to the mouse embryo assay (MEA) in Europe. In Australia, the Therapeutic Goods Administration (TGA) classified IVF culture media as class III medical devices in 2013. After a public consultation in November 2019 that considered the EU framework [21], classification rules in both regions are now very similar. However, Australian regulation and manufacturing requirements still differ mainly regarding MEA. MEA is used to assess embryotoxicity and functional performance of products for maintenance, culture, transfer, and cryopreservation of human embryos. Manufacturers must clearly state whether a one-cell or two-cell MEA was used and report results on product labelling. Additional requirements include bacterial endotoxin and pyrogen testing, biocompatibility, cytotoxicity and genotoxicity testing, non-clinical efficacy studies (including pregnancy rate testing with MEA), and clinical safety/efficacy data [22].

In the USA, the FDA classifies IVF culture media as class II medical devices [23]. MEA is recommended within quality assessment, and a “Guidance for Industry and Food and Drug Administration Staff” detailing MEA methodology was published in 2021 [4]. Endotoxin testing, sterilization validation, design specifications, labeling, biocompatibility, and clinical testing are also regulated. In China, culture media are class III devices under the NMPA. Requirements (once less stringent than in Europe or the USA) now include standards comparable to CE marking, such as ISO 13485 (or equivalent), but also MEA following the rule “YY/T 1434–2016 Medical devices for human in vitro assisted reproductive technology. In vitro mouse embryo assay”[24].

In the European Union, IVF culture media are class III devices under Directive 93/42/EEC (now replaced by Regulation (EU) 2017/745), and CE marking is mandatory. It might be reasonable to infer that Regulation 2017/745 or the 2015 European Commission document “Guidelines for Conformity Assessment of IVF and ART Products” [25] would require biological tests such as MEA for CE marking. However, a comprehensive search found no explicit reference to MEA in these documents.

Instead, Regulation 2017/745 sets broad safety and performance principles: devices must achieve the intended performance, be designed and manufactured to suit their purpose, and present risks outweighed by benefits. The manufacturer must specify and justify the level of clinical evidence needed to demonstrate conformity. The Regulation does not prescribe specific ART requirements or mandate particular testing methodologies. Regarding animal testing, recital 73 emphasizes the 3Rs and discourages unnecessary replication of tests. The EU framework also differs from medicines regulation: there is no European Medicines Agency premarket authorization or batch testing for devices. Market access depends on conformity assessment by a Notified Body (NB). In practice, this has likely produced a situation similar to the USA, China, and Australia, where MEA is commonly expected for embryotoxicity and functionality—not because EU rules explicitly require it, but due to regulatory inertia and the lack of alternatives offering equivalent information.

Chronopoulou and Harper [20] illustrated this de facto standard: all nine major Assisted Reproduction companies they examined (Cook Medical, Cooper Surgical-SAGE, FertiPro, Gynemed, Gynotec, Irvine Scientific, IVF online/LifeGlobal, ORIGIO, and Vitrolife) reported MEA testing of their IVF media on their websites. This raises the question of what MEA precisely entails. The following sections will define MEA, outline the procedures required to perform it, and examine its applications in the AR industry and beyond.

## The present: mouse embryo assay

### MEA concept

Vitrolife group (Assisted Reproduction industry section) defines MEA on their website as “a functional and toxicological bioassay utilised to detect toxicity and suboptimal compounds. It is a key tool to ensure quality of the media and devices that we manufacture. It also is a key factor that ensures the media is consistent and safe” [26].

The FDA, on the other hand, recommends that “MEA testing be used to assess the embryotoxicity of ART devices that have direct and/or indirect contact with gametes and/or embryos” [4].

Therefore, in the ART field, MEA is employed for the assessment of toxicity (embryotoxicity) and functionality of the products utilized for maintenance, culture, transfer, and cryopreservation of (human) embryos.

As for the procedure itself, it comprises the following steps:

### Step 1: Obtaining the one-cell or two-cell embryos

The recommendation from the FDA and other sources is to utilise hybrid mouse strains, with the CBA/B6 being the most frequently employed [4, 27]. However, a recent publication from Korchivaia et al. [28] has found that only a pooled sample of different mouse strains can be used for comprehensive media MEA testing. This is because hybrid strains with high reproductive efficiency can mask subtle differences between devices (e.g., culture media), and outbred and inbred strains can be too sensitive, which may result in embryos being delayed or degraded in culture media. In addition, Khan et al. proposed several years ago that outbred mice could be employed to enhance the sensitivity of MEA, as an alternative to inbred or hybrid mouse embryos [29].

Usually, female mice used for MEA are 5–6 weeks old, and they should be housed in a cyclic 12-h light/12-h dark schedule, although this may vary depending on the type of devices to be tested and their intended use. In regard to the number of embryos to be assessed in order to detect toxicity in devices with sufficient sensitivity, Punt-van der Zalm [27] et al. recommended 69 embryos per group, whereas the FDA proposes a minimum of 21 embryos [4].

**Fig. 1:**
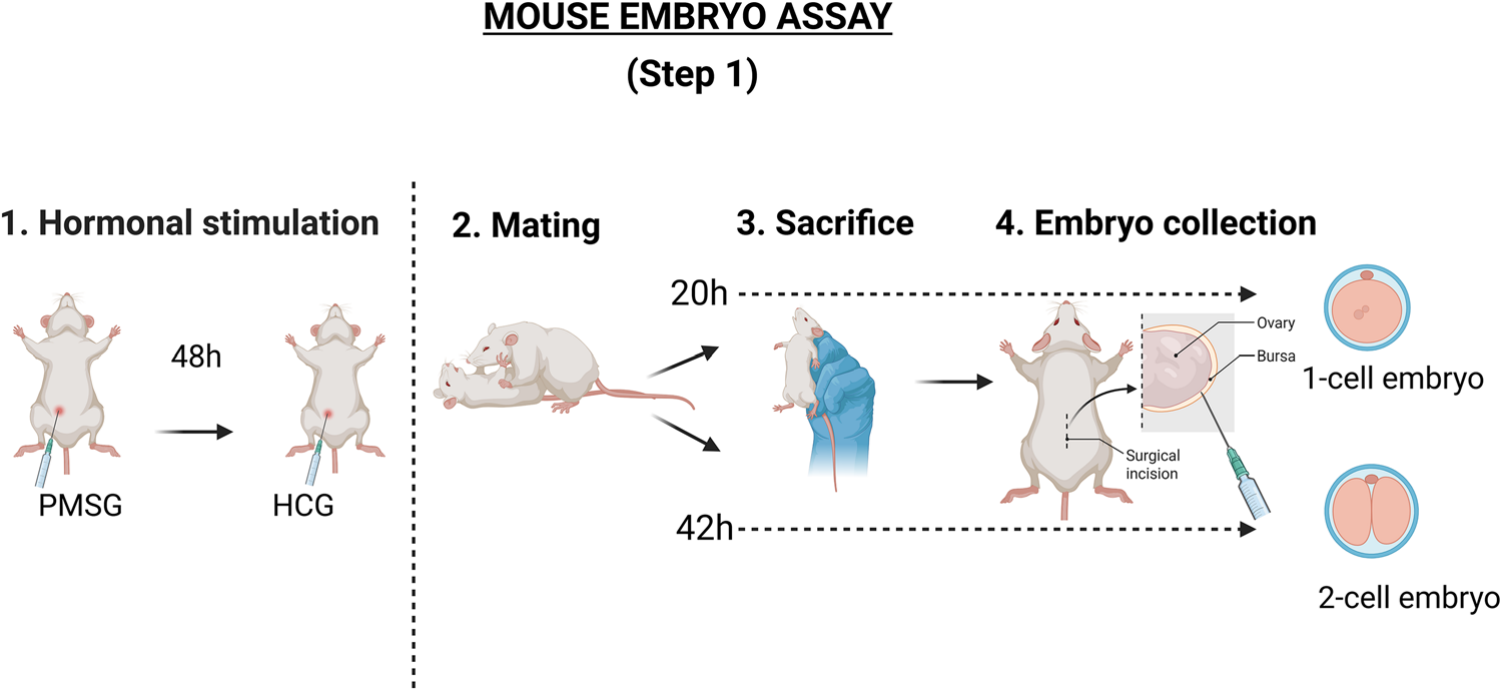
Standard protocol for obtaining 1-cell or 2-cell mouse embryos to be tested in the mouse embryo assay (MEA). Created in BioRender. Coy, P. (2026) https://BioRender.com/h21a420

Once the animals have been selected, the females are hormonally stimulated to ovulate via the administration of an intraperitoneal injection of pregnant mare’s serum gonadotropin (PMSG, typically 10 IU). This is followed, 48 h later, by the administration of another injection of human chorionic gonadotropin (HCG, typically 10 IU) (Fig. 1).

Each female mouse should be mated with one male mouse at the time of hCG injection and 24 h later mating should be confirmed by the presence of a vaginal plug.

The timing of the sacrifice of the females by cervical dislocation is contingent upon the assay to be developed, with the procedure occurring either 20 or 42 h after the hCG injection, depending on whether a one-cell or two-cell MEA is being utilized. Immediately following the sacrifice of the female mice, the abdominal cavity is opened under a stereomicroscope, the oviductal ampulla is visualised, and the cells are collected. This is achieved either by puncturing the oviducts (zygotes, 1-cell) or by flushing them with washing medium (2-cell). In the case of zygotes (1-cell MEA), it is essential to remove the cumulus cells by transferring the zygotes to a medium containing hyaluronidase. This is not the case for two-cell embryos, which are usually free of cumulus cells.

### Step 2: Embryo culture

**Fig. 2:**
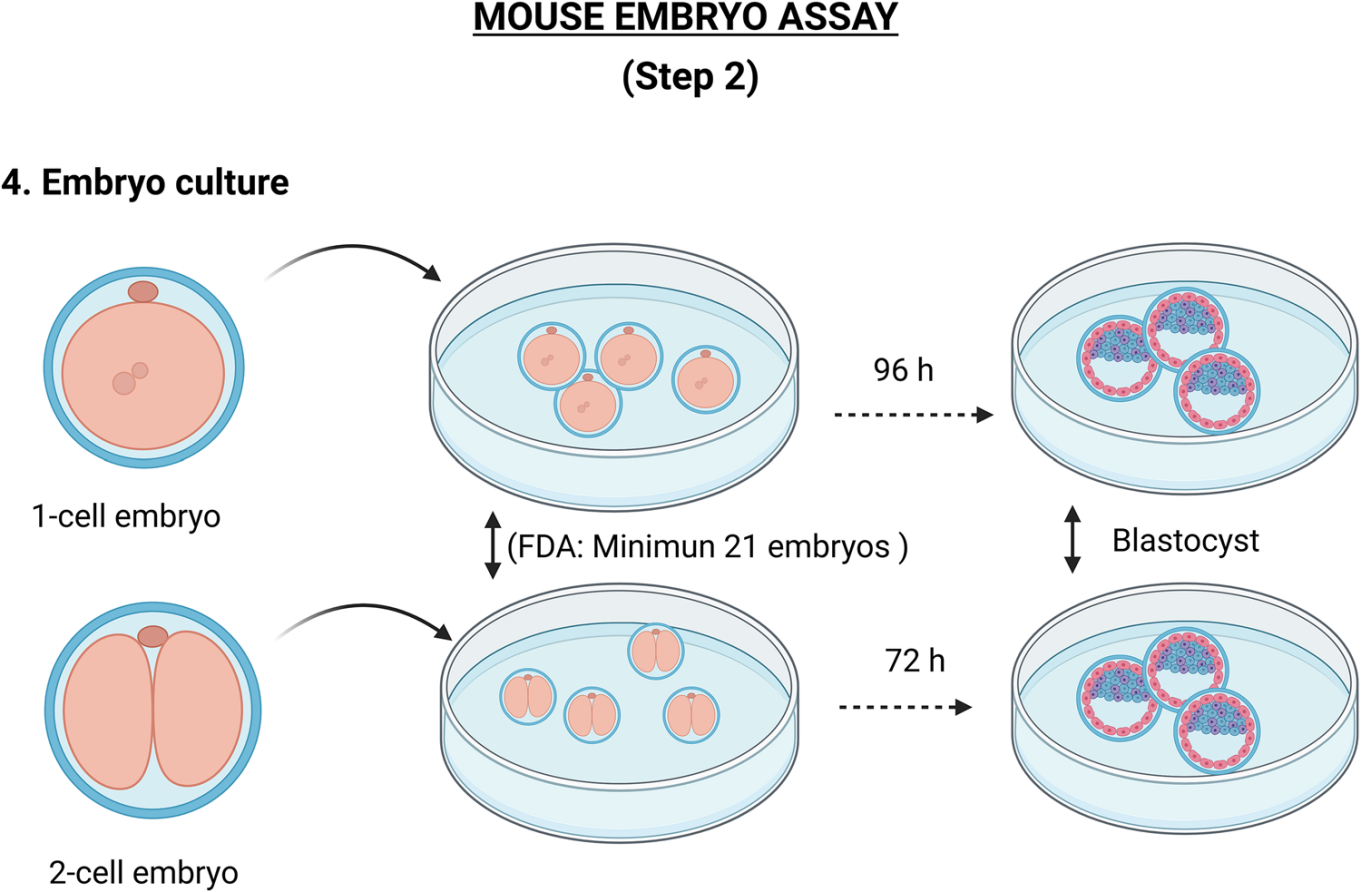
Standard protocol for the in vitro culture of 1-cell or 2-cell mouse embryos to be tested in the mouse embryo assay (MEA). Created in BioRender. Coy, P. (2026) https://BioRender.com/ev2gcss

In this step, embryos obtained in Step 1 are incubated in Petri dishes containing the corresponding embryo culture medium at standard conditions (usually 37 °C and 5% CO₂) for either 96 h or 72 h, depending on whether the assay carried out is the 1-cell or the 2-cell assay (Fig. 2).

Given the range of devices that may be subjected to testing, including those utilising culture media (for IVF, embryo culture, sperm selection, etc.), culture oil, plates or dishes intended for embryo culture, syringes, pipettes, and series of solutions employed sequentially (e.g., vitrification and warming of oocytes or embryos), it is essential to adjust the time of exposure in accordance with the specific use of the device. In most cases, this will entail monitoring the device until the conclusion of the intended incubation period under standard culture conditions.

### Acceptance criteria

It is possible for MEA endpoints to exhibit diversity and multiplicity. In general, the number of endpoints subjected to testing is directly proportional to the sensitivity of the test. In accordance with the recommendations set forth by the FDA, the following acceptance criteria, based on the embryo morphology, must be adhered to:
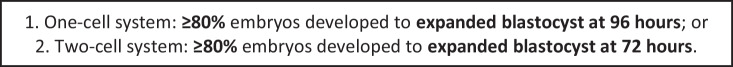


It should be noted that different companies consider these acceptance criteria to be the minimum requirements and add additional endpoints as a means of demonstrating the quality of their products. These endpoints may include the total number of blastomeres (total cell count), differential staining to assess the number of cells in the ICM and trophectoderm, blastocyst development at 78 h, morphokinetics, and the comparisons between strains of mice with different sensitivities to toxicity [14, 30].

### Uses of MEA

The use of MEA in the QC of various human IVF devices dates back to 1984, as documented by Ackerman [31, 32]. Since then, the use of MEA has become widespread, both in research and in the ART industry. For example, according to Vitrolife, one of the largest companies in the ART sector, the results of MEA testing for raw materials such as plastics, chemicals, or oil can give failure rates of up to 25%, 25%, and 60% respectively, hence the importance of good QC protocols [33].

In the context of Science, a comprehensive search of the PubMed database employing mouse embryo assay as a search term yields 48 papers (25/11/2025) whose primary theme or objective is delineated in Table 2.

**Table 2:** List of publications that include the text “mouse embryo assay” in PubMed® on 17/10/2024, classified by main topic/objective

Topic/objective of publication	References
Factors affecting MEA results (5)	Hendriks et al*.* [34] Korchivaia et al*.* [28] Martínez-Casado et al*.* [35] Mestres et al*.* [36] Nguyen et al*.* [37]
Testing formulations for human IVF (15)	Colazingari et al*.* [38] Fredrickson et al*.* [39] Gilbert et al*.* [40] Hentemann & Bertheussen [41] Hentemann et al*.* [42] Leonard et al*.* [43] McPherson et al*.* [44] Mestres et al*.* [36] Morbeck et al*.* [45] Morbeck et al*.* [46] Nguyen et al*.* [47] Quinn & Cooke [48] Schwarzer et al*.* [49] Viana et al*.* [50] Wolff et al [51]
Studying/diagnosing infertility problems (5)	Chen et al*.* [52] Chen et al*.* [53] Gómez-Torres et al*.* [54] Prough et al*.* [55] Vaquero et al*.* [56]
Testing toxicity (18)	Ainsworth et al*.* [57] Colucci et al*.* [58] Dubin et al*.* [59] Herrick et al*.* [60] Khan et al*.* [26] Lemeire et al*.* [61] Lierman et al*.* [62] Long et al*.* [63] Martínez-Casado et al*.* [35] Mestres et al*.* [36] Montoro et al*.* [64] Punt-van der Zalm et al*.* [27] Santos et al [65] Shi et al*.* [66] Terriou et al*.* [67] Van Merris et al*.* [68] Wolff et al*.* [51] Zeng et al*.* [69]
Opinions/discussions about MEA utility in lab and disposables QC (5)	Chronopoulou & Harper [20] Esfandiari & Gubista [30] Gardner et al*.* [14] Han et al*.* [70] Menezo et al*.* [71]
Proficiency testing (1)	Esfandiari & Gubista [30]
Non-related (2)	Delaroche et al*.* [72] Labied et al*.* [73]

In the industry, considering that medical devices for ART encompass any device that acts in a physical or mechanical manner during any IVF/ART procedure, the list of products to be tested is extensive. In addition to reproductive media (liquid and powder versions) and supplements added to media to enhance specific properties of the media (e.g., proteins, sera, antibiotics, etc.), pipettes, pipette tips, syringes, IVF tissue culture dishes, IVF tissue culture plates, gloves, and other vessels that come into physical contact with gametes, embryos, or tissue culture media are among the products to be tested. Moreover, MEA is today not only used in ART companies but also in the Life Sciences and Pharma industries, as exemplified in Table 3.

**Table 3:** Examples of companies engaged in activities related to AR, or not directly affiliated with the ART industry, utilising MEA in their quality control systems for different consumables

Company	Uses of MEA	Source
Cooper Surgical (including Origio, Life Global, RI) More than 600 products in more than 100 countries, from more than 27 brands Products include diverse medical devices such as needles, pipettes, catheters, culture media, dishes, etc	Quality Testing Across Our Brands: Every batch is tested by an independent laboratory using a validated mouse embryo assay (MEA). To maximize sensitivity: (i) one-cell zygotes are cultured to fully expanded blastocysts (FEB); (ii) a simple culture medium is used to slightly “stress” the embryos; (iii) protein-free culture medium is used so that toxicity is not masked by protein; (iv) embryos are directly exposed to pipette tips for the entire 96-h culture period Product example: Wallace® dual lumen oocyte recovery systems: Quality Control Testing Mouse Embryo Assay (MEA)	https://www.coopersurgical.com/healthcare-providers/support-compliance/quality-certifications/ (CooperSurgical) [74] https://www.coopersurgical.com/product/wallace-dual-lumen-oocyte-recovery-systems/ (CooperSurgical) [75]
Ferti-Pro Manufacturer of in vitro diagnostics and cell culture media used in assisted reproductive technologies and the diagnosis of male infertility	All 10 culture media contacting with oocytes or embryos are tested by mouse embryo assay: 80% blastocysts after 96 h culture Product example: Ferticult™ IVF medium	https://fertipro.com/2019/12/10/ferticult-ivf-medium/ (Fertipro) [76]
Vitrolife Raw materials Plastic disposables Media Contact material 142 products embryo tested	“Vitrolife has developed the most sensitive MEA protocols. These assays are capable of detecting toxic and sub-optimal raw materials, media, and contact materials. The MEA from Vitrolife is sensitive enough to identify subtle problems that will also lead to impaired human embryo development.”	https://www.vitrolife.com/products/labware/dishes/ (Vitrolife) [77] https://www.vitrolife.com/search/?content=Products&q=embryo%20tested&page=11 (Vitrolife) [78]
Fujifilm-Irvine Scientific	“Our proprietary genetic mouse embryo assay (MEGA) to identify embryotoxicity at an epigenetic level and provide a more sensitive, relevant testing method of critical raw materials than 1-cell MEA alone”	https://www.irvinesci.com/art-quality(Irvine (Scientific) [79]
Cook Medical	51 reproductive health-related products, including pipettes, catheters, needles… No culture media or culture plates found	https://www.cookmedical.com/ (CookMedical) [80]
	No data on QC found on the website	
Genea Biomedx (Genea Biomedx (formerly Cook, then Sydney IVF and now Genea)	No information about media characteristics nor QC are available at the website	https://www.geneabiomedx.com/ (GeneaBiomedx) [81]
Gynemed Development, manufacture and distribution of high-quality medical products for reproductive medicine	MSDS for culture media indicate: “Extensive data on Mouse Embryo Assay have demonstrated that the medium is not toxic” Example of QC for Mineral oil for covering medium: MEA (Blastocysts after 120 h in culture in %): ≥ 80) Example for other consumables indicates “MEA and LAL” in QC	https://gynemed.de/en/products/sicherheitsdatenblaetter/ (Gynemed) [82] https://gynemed.de/en/products/gm501-mineral-oil/(Gynemed) [83] https://gynemed.de/en/products/embryo-transfer-katheter-dreiteilig/ (Gynemed 2) [84]
Gynotec/CCD/Kanin Laboratory Dedicated to provide high quality and best priced ART medical devices	Mouse embryo assay: ≥ 80% Moreover “A mouse embryo assay is routinely performed as part of the batch release criteria of HAS.” MEA for Culture media example: IVF-basics-HTF MEA for other consumables example: aspiration needle:	Summary of Safety and Clinical Performance at https://gynotec.nl/ivf-basics-htf-0-4-hsa/ (Gynotec) [85] https://gynotec.nl/overview-oocyte-retrieval/ (Gynotec) [86]
Nidacon International	MEA for medical devices or related products used in ART	https://nidacon.com/why-nidacon/#about (Nidacon) [87]
“Develops, manufactures, and markets medical devices and solutions for ART”	**Mouse Embryo IVF Assay for Media**	
Microm UK Ltd, part of the Calibre Scientific group Products for assisted reproduction, embryology and semen analysis	MEA for culture media: ≥ 80% blastocysts after 96 h incubation	https://investor.calibrescientific.com/brands/microm: https://microm.co.uk/products/ferticult-ivfmedium/ (Microcom) [88]
Kitazato Dibimed	Culture media and other consumables Gamete buffer medium 1-cell MEA (blastocysts after 96 h): ≥ 80% after 1 h of exposure (zygote stage) OPU Needle: MEA (1-cell, % blastocysts after 96 h): ≥ 80%	https://kitazato-ivf.com/wp-content/uploads/2024/04/IFU_Gamete-Buffer_2024.pdf (Kitazato) [89] https://kitazato-ivf.com/wp-content/uploads/2024/04/IFU_OPU-Needle_2024.pdf (Kitazato) [90]
Embryotech	MEA offered as a service for embryotoxicity and functionality testing	https://embryotech.com/ivf-mea (Embryotech) [91]
Monash Biotech	MEA used to test a variety of micropipettes	https://www.monashbiotech.com/es/blog/the-mouse-embryo-assay-mea-test-a-gold-standard-for-human-in-vitro-fertilization-ivf-quality-control/ (Biotech) [92]
ElliosBioTek	MEA included in the QC of all the products related to ART (catheters, needles, tubes, etc.)	https://ellios-biotek.com/engagement-qualite/ (BioTek) [93]
WillcoWells (MEA done by EmbryoTools)	MEA is used in dishes and glass wells	https://willcowells.com/certificates-willcowells (WillCoWells) [94]
Corning Life Science	Plates and dishes “Each lot of Falcon IVF product is tested for embryotoxicity using the one-cell mouse embryo assay. A minimum of 75% of both test and control embryos must reach the hatched and/or expanded blastocyst stage in order for our products to be deemed nonembryotoxic and acceptable for product release.”	https://www.corning.com/emea/en/products/life-sciences/resource-library/search.html?search-tag=%7B%22k%22%3A%22hierarchicalfacet_applications_en_gb_s%22%2C%22v%22%3A%22In+Vitro+Fertilisation%22%7D&q=*&initialResultType=resources (Corning Life Sciences) [95]
Sunlight Medical	Micropipettes for ICSI, embryo biopsy, assisted hatching, MEA: > 80% blastocysts	https://www.sunlightmedical.net/certanalysis/ (Sunlight Medical) [96]
SIGMA (MERCK)	43 products are labels as “suitable for embryo cell culture,” including Hepes, sodium pyruvate, mineral oil, glucose, antibiotics, culture media, enzymes, etc In the specification sheets from SIGMA products, “suitable for embryo cell culture” means: Embryo Test Single cell embryos reach blastocyst stage 80% Example: W1503. Water suitable for mouse embryo cell culture. H20: “embryo transfer pass”	https://www.sigmaaldrich.com/ES/en/search/embryo-cell-culture?focus=products&page=1&perpage=30&sort=relevance&term=embryo%20cell%20culture&type (Sigma Merck) [97] https://www.sigmaaldrich.com/ES/es/specification-sheet/SIGMA/W1503 (Sigma-Merck) [98]
Charles River	MEA is used for toxicity and functionality testing of media, labware, disposables or any device that may come into contact with gametes or embryos	https://www.criver.com/products-services/research-models-services/genetically-engineered-model-services/embryology/mea-testing?region=3696 (River) [99]

In consideration of the data presented in Table 3, it becomes evident that thousands of devices are tested annually using MEA. It thus appears obvious that a considerable number of mice are required to conduct these tests. Furthermore, it is indisputable that thousands of cadavers and organic residues will be delivered from the facilities where the animals are bred. This naturally raises the question of whether it is possible to estimate the number of animals sacrificed and the amount of waste generated by MEA on an annual basis, and whether alternatives currently exist to avoid both.

### Estimated number of animals sacrificed for MEA: environmental costs of laboratory animal facilities

The number of animals used in MEA could be estimated from three main sources: private industry, government laboratories, and public research institutions. Of these, the last likely accounts for the fewest animals but offers the best opportunity to obtain transparent, realistic data.

Mice are treated differently in the USA than in Canada, the UK, Switzerland, and the EU because the USA reporting under the Animal Welfare Act (AWA) excludes mice, rats, and fish. As a result, the USA lacks a comprehensive system for collecting statistics on these species, making realistic estimates harder than in other regions [1].

In 2021, Larry Carbone attempted to estimate animal use in American laboratories by extrapolating from the United States Department of Agriculture (USDA) data on AWA-covered mammals and from institutional annual reports [1]. He obtained rat and mouse numbers from 16 of the top 30 National Institute of Health grant recipients (11 via public records requests and 5 voluntarily). Comparing these data with USDA reports, he found that rats and mice represented 99.3% of mammals used annually at these large institutions. Applying this proportion to the USDA national total of 780,070 animals for 2017–2018, he estimated that ~ 111.5 million rats and mice were used annually in 2017–2018. Without official data on quality control or toxicity testing, however, the number of mice used specifically for MEA in the USA remains almost impossible to determine.

The situation is more transparent in Europe, Canada, and Australia. Openness initiatives are in place in Belgium, France, Germany, the Netherlands, Portugal, Spain, Australia, and New Zealand, involving universities, research institutions, and pharmaceutical companies [100]. As early as 2016, a European Partnership for Alternative Approaches to Animal Testing (EPAA) panel agreed on the need to implement validated alternatives and to harmonise validation criteria internationally, while calling for faster adoption, more flexible guidance, and clearer scientific method descriptions [101].

The work carried out by the EPAA soon bore fruit, as reflected in EU statistics. In 2019, the European Commission’s report (based on data from 2017) revealed that 72% of animal use was for research purposes (including 37% for basic research), 13% for regulatory purposes and 6% for routine production [102]. In 2017, 2.18 million animals were used for regulatory purposes: Of these, 52% were used for quality control (79% of which was for batch safety and potency testing, where MEAs may be included), 39% were used for toxicity and other safety tests, including pharmacology (where MEAs may be included, with reproductive toxicity being the most common reason for testing), and 9% were used for other efficacy and tolerability testing (with MEA involvement unclear) [102]. Importantly, regulatory use fell to 1,103,612 in 2022 (approximately 50% lower than in 2017), representing a 32% decrease compared to 2018 (1,622,816) and a 16.2% decrease compared to 2021 (1,317,252).

**Fig. 3:**
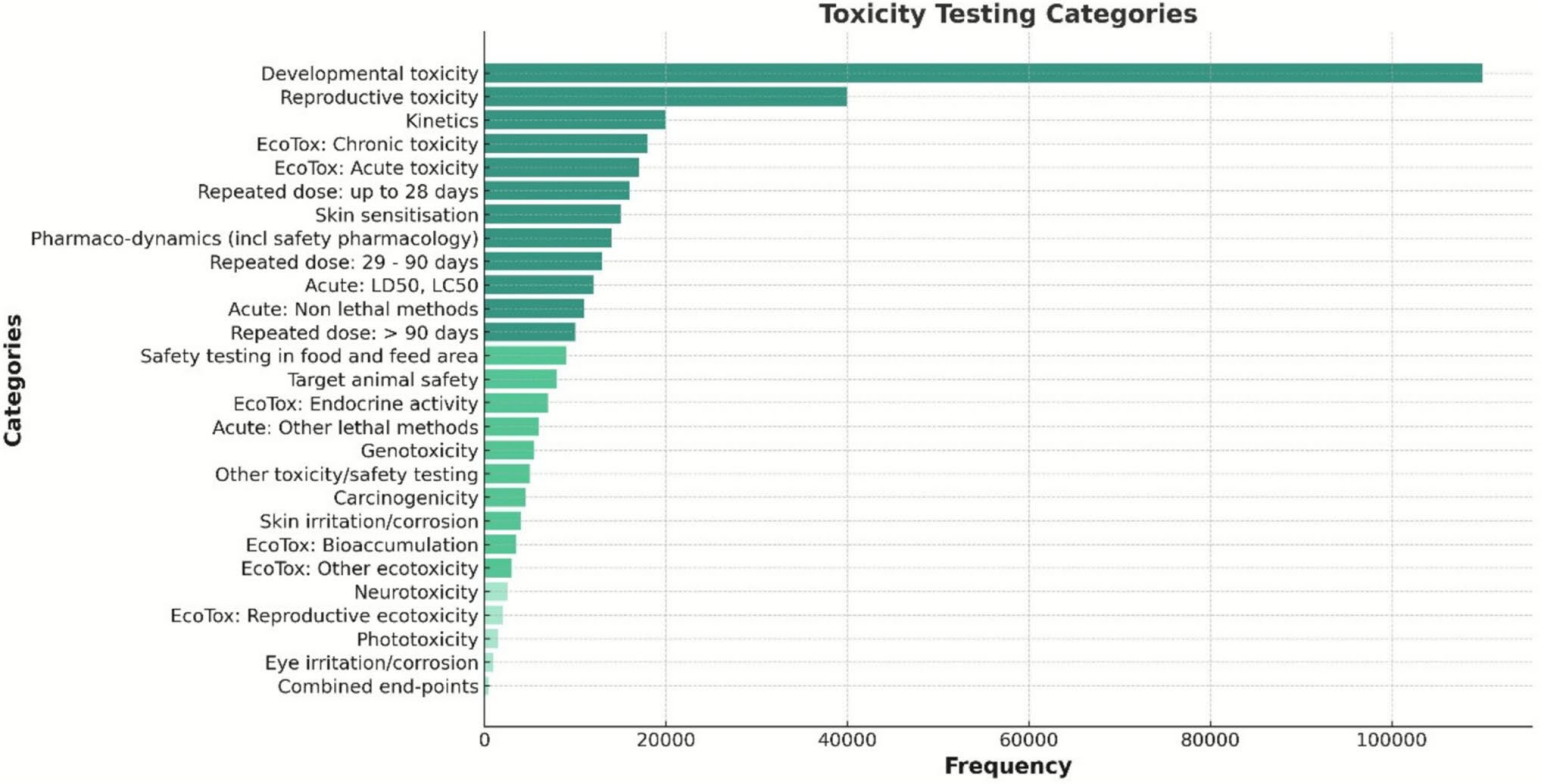
Toxicity and other safety testing including pharmacology by type of use in 2022 *(adapted from the Summary Report on the statistics on the use of animals for scientific purposes in the Member States of the European Union and Norway in 2022)* (Council of European Commission 2022)

Of the approximately 1.1 million regulatory uses in 2022, 51.8% were for quality control, 45.3% were for toxicity and other safety tests, including pharmacology, and 2.9% were for other efficacy and tolerability tests. Within the toxicity and other safety tests (which should include MEAs), the most common categories were developmental and reproductive toxicity [103] (Fig. 3).

Contrary to what might be expected, the number of animals used is not directly proportional to the number of breeding facilities because many bred animals are never used. In 2022, more than 9 million animals across the EU were bred but not used [104]. Combining ~ 7 million used with 9,572,759 produced but not used (7,686,858 mice) suggests roughly 16 million animals bred annually. Using official 2022 statistics, one can estimate ~ 11.7 million mice bred for scientific purposes in the EU and Norway (about 4.0 million used in procedures and ~ 7.7 million bred, killed, and not used) [105, 106].

A preliminary survey suggests a single European facility may breed ~ 3 million rodents per year [107]. In the USA, USDA Animal and Plant Health Inspection Service notes that among 8751 regulated entities in 2022, ~ 1,055 were registered research facilities [108]. The COSCE (*Confederación de Sociedades Científicas de España*) Transparency Agreement reported 496 facilities across eight countries in 2022, plus 27 in New Zealand, and the Association for Assessment and Accreditation of Laboratory Animal Care accredits over 1,100 organisations in 50 countries/regions [109].

If estimating the global number of facilities is difficult, quantifying their environmental footprint is even harder. Using ~ 11.7 million mice bred annually in Europe and an average mouse weight of 20–40 g yields ~ 351 tons of carcasses to manage each year. If MEA required ~ 300,000 mice worldwide annually (as an estimation), this would represent ~ 9 tons of carcasses. Disposal methods for this waste include rendering, landfill, and incineration; some facilities incinerate on-site while others rely on commercial services [110]. This means that animal research facilities generate ignitable, corrosive, reactive, and toxic wastes and emit air pollutants such as nitrogen dioxide, sulfur dioxide, particulate matter, and carbon monoxide [111]. Energy demands are also higher than in standard labs: air exchange rates can reach 20 air changes per hour versus 12 in typical laboratories, with additional burdens from space, lighting, ventilation, humidity control, and barrier protection [111].

Overall, the carbon emissions of animal testing are an increasingly pressing but “hidden” environmental impact due to limited transparency compared with livestock production [112–114]. The author noted the scarcity of reliable data and the absence of PubMed publications explicitly quantifying breeding facilities or their environmental costs. Encouragingly, organisations such as Lambda are developing animal-free alternatives for preclinical or quality control applications [115]. For MEA specifically, global innovation should be accelerated in the most cost-effective and rapid way possible, since the 3Rs and animal rights are often not considered in this context. Moreover, using mice is difficult to justify when alternative species could provide comparable results using slaughterhouse by-products, as discussed below.

### The animal rights and the 3Rs principle

It was in 1796 that probably the first publication appeared suggesting the need for common laws recognising that animals need to be cared for by humans [116]. Almost a century later, the term “animal rights” was developed in Henry S. Salt’s book “Animals’ Rights: Considered in Relation to Social Progress” [117]. But it took another two centuries before the Universal Declaration of Animal Rights was proclaimed in Paris in 1978 [118]. One of the key proclamations of the Declaration was that “the killing of an animal without justification is prohibited. If the killing of an animal is justified, it should be instantaneous, painless and should not provoke anxiety.” Although the MEA tests meet the second part of this statement, its justification would be questioned if alternatives with similar results were provided.

The Declaration did not achieve the expected level of global commitment officially, yet society continued to advance, and animal welfare organisations proliferated on a global scale. Indeed, a new Universal Declaration on Animal Welfare is currently under proposal for adoption by the United Nations, with a seemingly greater probability of endorsement and longevity [119].

In any case, evidence of the growing societal awareness in this area can be found in the fact that nine countries have enshrined language protecting animals in their constitutions, despite acknowledging the need for animal use in different fields [3]. Furthermore, most countries have now adopted at least basic legal prohibitions against animal cruelty, and only 11 countries worldwide have no laws protecting animals [3].

The positions of animal welfarists may seem extreme to some people, but what does seem universally accepted and included in the laws and regulations of most countries is the principle of the 3Rs.

Under EU Directive 2010/63/“researchers must use non-animal alternative methods whenever they are possible. Every research project that makes use of animals must be authorised before it can start, and it must include a scientific explanation of why animal research is needed in the project.”

This statement serves as an exemplar of the successful implementation of the 3Rs in the EU. The principle was first articulated by Russell and Burch in 1959 [2], but it was not until it was incorporated into the majority of official regulations pertaining to animal experimentation globally that it became a widely accepted practice. To illustrate, the European Community enacted its inaugural legislation (European Directive 86/609/EEC) in November 1986. While this legislation did not explicitly mention the Three Rs, it did require member states to implement national legislation that put into practice a significant proportion of the Three Rs principles [120]. Currently, things have evolved and the number of scientific societies, governments, private companies, civil movements or research institutions that have adopted the 3Rs philosophy has only increased, representing the paradigm of correct human behaviour towards animals, although not exempt from the need for updates.

The 3Rs stand for Replacement, Reduction, and Refinement. Each of these three words offers animals in general, and laboratory animals in particular, the possibility of not being used in experiments at all, of being used but in smaller numbers than otherwise expected, and, when used, of being treated with “humanity,” i.e., of minimising their suffering as much as possible. In other words, the 3Rs represent the ethical treatment of laboratory animals.

Despite a general consensus that this principle is widely accepted and that there is a downward trend in the use of animals, it is evident that millions of animals are still being used globally [120] (as previously discussed). This indicates that there is much to be done to achieve the goal of reducing the number of animals used in research. The ethicality of this practice is not in question when it is for the purpose of saving human lives. Nevertheless, it is debatable whether all current applications are genuinely indispensable and whether alternative techniques can supersede the existing ones for particular objectives, as exemplified by MEA. Under this perspective, one could wonder if the *animals used annually for MEA test could not be effectively Replaced*…

One might posit that the slow progress in reducing the use of animals for purposes such as MEA is due to the difficulty in developing scientific methods that can adequately mimic the complexity of this assay. However, following the development of ART in a range of mammalian species, particularly in bovines [50], it is unclear why there have been no significant efforts to replace mice gametes and embryos with those of livestock species, given the ready availability of this organic material as a by-product of the meat industry. This argument will be developed in the last section of this article.

Another argument to continue using MEA can be the belief that the use of the MEA is mandatory under current regulatory standards, particularly in countries outside Europe. However, a careful read of the FDA guidelines [4] shows that this is not the case. Indeed, the footnotes of the guidance state that “FDA supports the principles of the “3Rs,” to reduce, refine, and replace animal use in testing when feasible. We encourage sponsors to consult with us if it they wish to use a non-animal testing method, they believe is suitable, adequate, validated, and feasible. We will consider if such an alternative method could be assessed for equivalency to an animal test method.” Even more, in the same guidelines is clarified that “The use of the word “should” in Agency guidance means that something is suggested or recommended, but not required.” Finally, a recent article in Nature describes how FDA has proposed the goal of phasing out animal toxicity testing in 3–5 years [5].

## The future: the bovine embryo assay (BEA) as an alternative to MEA

Since its inception, the number of voices proposing improvements or changes to MEA testing in the ART industry and within the scientific community has been increasing, particularly since the publication of the FDA Modernization Act 2–0 which indicates that “animal testing should be phased out with the exception of appropriate allowances” [121]. However, there has been a paucity of implementation for realistic alternatives.

One of the primary criticisms of the MEA is its lack of standardisation, which consequently impacts the consistency of the results. The protocols outlined in the FDA Guidelines [4] are not universally defined, and ambiguity exists with regard to subjective analysis of morphology in developing embryos, culture conditions, media, O_2_ concentration, size of medium drop, number of embryos in each drop, pH, osmolality, and other factors. This variability among laboratories can introduce inconsistencies and potentially compromise the reliability of the results. A 2018 survey by Delaroche [72] of several leading IVF device manufacturers revealed significant differences in MEA methodology and thresholds for toxicity testing. Some of these parameters influence test sensitivity. In the authors’ words, their “study confirms the high degree of heterogeneity of the embryotoxicity tests performed by manufacturers when validating their IVF disposable devices. Future recommendations are urgently awaited to improve the sensitivity and reproducibility of embryotoxicity assays over time.”

A number of eminent scientists have also expressed reservations about MEA, citing various reasons. One such contention refers to the evident disparity in the reproductive physiology of mice when compared with that of humans [7]. Indeed, mice ovulate multiple oocytes per cycle (≈10) while human is a single ovulation species. The final stages of oocyte maturation appear to be subject to more stringent regulation in humans, where a minimum follicle size is required for in vitro maturation to occur [7]. Furthermore, the developmental timeline of the mouse embryo is characterised by the attainment of the morula stage on day 2.5, followed by the progression to the blastocyst stage by day 3.5–4 [122]. Conversely, the human embryo reaches the morula stage on day 4, and the blastocyst develops by day 5 [123]. With regard to embryonic genome activation (EGA), this occurs at the 4–8 cell stage in the human [124] and the 1–2-cell stage in the mouse [125]. Finally, it is noteworthy that the gestation period is shorter in mice, spanning less than 1 month, and longer in humans, extending to approximately 9 months.

As Chronopoulou and Harper [20] pointed out, there are other reasons why people have criticised MEA. These include the fact that the results can be different depending on the strain [59, 126, 127], the ability of mouse embryos to develop into blastocysts when cultured in tap water without any protein supplement [128, 129], and the finding that mouse embryos that lost one blastomere at the 2-cell stage and therefore had reduced inner cell mass (ICM) developed into blastocysts and achieved implantation [130]. Nonetheless, the primary impediment to the continued utilisation of MEA for regulatory and testing purposes within the domain of ART, as perceived by the author, is ethically grounded. Contemporary alternatives have emerged that no longer substantiate the continued employment of MEA.

Among them, the use of bovine model [7], functional molecular biomarkers [41], time-lapse imaging, human embryonic stem cell-based assays [91], “organ-on-a-chip” technology, organoids, digital artificial intelligence, and machine learning [121] have been proposed. Of course, the most similar test to MEA from all the above referred is that using the cow as an animal model, that is the bovine embryo assay (BEA). The advantages of this model begin with the similarities between the reproductive cycle of the cow and the woman, being the cow a single ovulation species, where the final stages of oocyte maturation are similarly regulated to humans [7], the developing embryo reaches morula stage at day 5, blastocyst stage at day 7–8, EGA occurs at 8–16 cells [131], and the gestation takes 9 months as it happens in humans. Also, the availability of biological material is unlimited, since the oocyte donors are not experimental animals bred with the only purpose of being hormonally stimulated, mated, and sacrificed to collect their oocytes, zygotes, or embryos, but animals come from the dairy or meat industry, are bred to provide food for the human consumption and sacrificed in slaughterhouses under strict sanitary and welfare controls.

Two major problems can be deduced from the bovine model: the variety of breeds and genetic background at abattoirs, and the lack of standardised protocols. The former can be regarded as a benefit, however, as referenced in the recent studies by Korchivaia et al. [28], which propose that only a pooled sample of different mouse strains can be used for comprehensive media MEA testing. Interestingly, despite the significant variations in slaughterhouse materials and embryo production protocols between laboratories, the blastocyst yield at the end of the culture period remains consistent at 30–40% [132–135]. This figure can therefore be used as a reference standard for comparison in an embryotoxicity assay. The second issue may be resolved through the implementation of appropriate laboratory practices, such as those outlined in ISO 17025:2017, thereby ensuring the reliability and reproducibility of the results obtained.

Given the increasing number of laboratories and industries producing bovine embryos worldwide (nearly 1.6 million in vitro-derived cow embryos were transferred in [50], the development of standardised and authorised BEA protocols, accepted by regulatory agencies in each country, does not seem utopian. Efforts to achieve this goal are already underway in our laboratory and others, with comparable data between MEA and BEA [6]. Among other advantages (see Table 4), BEA enables the toxicity of devices and culture media to be assessed at various stages of the in vitro embryo growth process, from the collection and manipulation of oocytes to in vitro fertilisation, or development until the two-cell stage, the point at which standard MEA begins. Therefore, unlike the standard MEA described by FDA, not only is the sensitivity of two-cell embryos to developing into blastocysts in a specific culture medium or device assessed, but also the sensitivity of oocytes and spermatozoa to being manipulated or fertilised in such devices.

**Table 4:** Merits and drawbacks of replacing the mouse embryo assay with the bovine embryo assay when assessing the functionality and toxicity of medical devices in the assisted reproduction industry

Advantages	Disadvantages
**•**Single offspring (like humans) •Long reproductive cycle (21 days) and gestation (9 months) •No need of animal sacrifice to run the tests •More similar to humans in terms of biochemical and intrinsic paternal and maternal regulatory processes •Similar regulation of the final stages of oocyte maturation to humans •Embryo development chronology and EGA more similar to human •Gastrulating/neurulating processes are closer to humans •Unlimited availability of biological material •Big group size, hence high statistical power and less variability across groups •Obtaining semen is an easy process thanks to the numerous specialized companies dedicated to commercializing it for artificial insemination •No need of the establishment of dedicated animal care facilities or the allocation of extra personnel for this purpose •Bovine embryos are more sensitive to environmental conditions (concerns about mouse embryo sensitivity) •Mouse embryos not requiring amino acids to develop to the blastocyst stage, in contrast to bovine and human embryos •Mouse embryos are less sensitive to and recover more easily from changes in pH than either human or bovine embryos •Non ethical issues •Greater social acceptance •Contribution to sustainability and organic waste recovery	**•**Single offspring (more difficult to perform long term studies with the progeny) •Long reproductive cycle (21 days) and gestation (9 months) •High background pregnancy loss •Limited availability of pure breed animals •Expensive for offspring studies •Less historical control data and laboratory experience/capability •Highly variable age, weight and pregnancy history at the start

Some other advantages and disadvantages of BEA versus MEA are shown in Table 4.

## Conclusions

1. Assisted reproduction technologies (ART) encompass a growing set of tools whose demand is increasing exponentially as clinical indications continue to expand and diversify. Today, millions of individuals worldwide rely on ART to address fertility challenges.
2. With the global proliferation of infertility clinics, the implementation of robust quality management systems and regulatory controls has become increasingly necessary. The mouse embryo assay (MEA) is currently the reference test used to assess functionality and embryotoxicity in ART devices. It is estimated that hundreds of thousands of mice are sacrificed annually for this purpose. However, MEA has been subject to growing criticism due to concerns about its sensitivity, lack of standardization, and ethical implications.
3. A thorough review of international regulations reveals that the use of MEA for toxicity and functionality testing of ART-related medical devices is not mandatory in most regions. On the contrary, many regulatory agencies actively promote the use of alternative methods that reduce or replace animal use, in line with the 3Rs principle (Replacement, Reduction, and Refinement) and the broader goal of advancing animal welfare.
4. This article proposes the gradual replacement of MEA with the bovine embryo assay (BEA) and recommends that BEA be incorporated into the regulatory frameworks of relevant institutions worldwide. BEA does not require the use of laboratory animals, relying instead on by-products from slaughterhouses, where animals are already processed for food production. Coordinated efforts among research institutions, cattle reproduction laboratories, regulatory bodies, and ART device manufacturers will be essential to support this transition and to establish BEA as a reliable, ethical, and scientifically robust alternative to MEA.

## Data Availability

No datasets were generated or analysed during the current study.
